# “Scarless world or scar-less world”: expedition on new perspectives on management of post-burn hypertrophic scar

**DOI:** 10.1186/s41038-016-0049-5

**Published:** 2016-05-24

**Authors:** Cecilia W. P. Li-Tsang

**Affiliations:** Department of Rehabilitation Sciences, The Hong Kong Polytechnic University, Hung Hom Hong Kong, China

Hypertrophic scar has always been the top priority of post-burn intervention. Specialists from multiple disciplines have dedicated enormous efforts expanding the knowledge in understanding the etiology of hypertrophic scar formation and establishing the scientific evidence of effective management of hypertrophic scar. The devotion and pursuit of these scientists and clinicians has been especially meaningful for the population in Asia since it has been well proven that hypertrophic scar was endemic to the Asian population comparing to the Caucasians. Besides early intervention, functional outcomes and long-term quality of life for people with extensive burn injuries have been investigated by different researchers and further substantiate the importance of long-term continuum of rehabilitation programs [1, 2].

In Asian countries such as China, management of hypertrophic scar is mainly confined to surgical removal of scar causing contracture, deformities, and/or disfigurement. Early and active scar management is rarely practiced soon after surgical implementation. Thus, functional outcomes are expected to be poor. In the past two decades, burn specialists have begun to realize the importance of functional outcome of patients with massive burns in China [3]. New evidence regarding the quality of life of patients with extensive burn injuries identified a poorer quality of life with prolonged physical and psychological problems. Fortunately, there is a positive trend of gradual improvements noted but slow [2]. As the socio-economic boost of the society and the advancement in the quality and standards of medical care, the ultimate goal of post-burn management has been shifting from survival to functional recovery, since both the patient and the health care professional gradually strive for a better quality of life with social re-integration.

The thorough understanding of etiological and molecular basis of hypertrophic scar formation has been considered as the foundation of effective prevention and management of hypertrophic scar. In this special issue, Zhu, Ding, and Tredget [4] conducted comprehensive review on the molecular basis of hypertrophic scars, highlighting the roles of cytokines, growth factors, and macrophages via chemokine pathways. In Tan et al.’s recent article [5], novel technology of isobaric tags for relative and absolute quantitation (iTRAQ) was applied in order to advance our exploration on the various mechanisms and cellular signaling pass ways of hypertrophic scar formation. The comprehensive investigation identified multiple up-regulated and down-regulated proteins, which might serve as indicators for future management strategies.

Along with the advocacy of evidence-based medicine, international burn society has gradually recognized the importance of research evidence in guiding clinical practice. Several international guidelines have been published in recent years based on extensive literature reviews. Despite the limited research design and inadequacy of clinical data available, consensus in several areas on scar management was finalized and new intervention strategies could be explored [6, 7]. Interestingly, despite the relatively adequacy of the literature supporting the effectiveness of silicone gel and emergence of all the alternative interventions, a survey of burn centers across North America and Australia suggested that the most common intervention are still splinting, postoperative ambulation, conditioning, scar massage, and use of compression garments [8].

In accordance with the International Society for Burn Injuries, the first *Guidelines for Burn Rehabilitation* in China has been published by the Chinese Burn Association, Chinese Association of Burn Surgeons, and The Chinese Burn Care and Rehabilitation Association in 2015. In this guideline, the concept of early intervention was strongly advocated to all practicing therapists in burn management. For scar management, pressure therapy, silicone gel sheets, massage, and intralesional injection were suggested [9].

China was not the only country that recognized the predominance of hypertrophic scar in Asian population. In 2013, a guideline of scar management especially targeting Asian patients was published based on literature review and a panel of experts’ opinions. The guideline not only stressed on the importance of early intervention, but also advocated on scar prevention. Unlike the rather conservative Chinese guidelines, a wide variety of novel modalities were introduced and evaluated on their clinical efficacy, namely, intralesional steroid injections, radiation therapy, and intralesional 5-fluorouracil injections. Laser treatment and surgical removal were also proposed as the last resort on scar management. Unlike international guidelines, this panel of experts did not recommend pressure therapy as the mainstay of scar management [10].

One of the possible reasons for the discordance of aforementioned different expert panels may be due to the diverse and subjective scar measurements on the clinical outcomes of various intervention modalities. Fortunately, the concept of standardized objective scar assessments has been strongly advocated in the past decade from research institutions to clinical settings. In this special issue, a systematic review which featured exclusively on objective scar measurements was presented by Lee and his team in UK [11]. Despite the escalation in the quantity of objective scar assessments, inconsistent evidence of the quality of these objective scar assessments demands further investigations and refinements. Nonetheless, the recommendations proposed in this paper could offer us various promising options to measure different dimensions of scar objectively and accurately. Thus, more evidence could be scientifically supported on different intervention regime.

Another obstacle towards consensus may be due to the inadequate and the inconsistent clinical evidence available, especially for the expanding numbers of novel treatment methods. This time, two categories of scar treatment will be addressed, one is intralesional injection and another is natural therapeutics. Intralesional injection of various therapeutic agents including triamcinolone, bleomycin, 5-fluorouracil, verapamil, was among the most popular options in scar treatment in the recent decade. However, Trisliana Perdanasari et al. [12] revealed that extra discretion was required for decision making in using intralesional injection due to sparse evidence at present. Comparing to intralesional injections, natural therapeutics gained reputation from practitioners and researchers due to non-invasive nature. In the review conducted by Rolfe et al. [13] both plant-based product and nonplant-based therapeutics were explored and evaluated based on in vivo and in vitro experiments. Clearly, these fascinating fields show great potential which enchant us for further exploration.

In addition, preventive measures against hypertrophic scarring were not only taken after the wound closure. It is widely accepted that prolonged wound healing would induce abnormal scar formation; thus, one key factor to prevent formation of hypertrophic scar is to facilitate early wound closure. Innovative technology of tissue engineering is important to enhance early wound healing. Two papers on comprehensive review of hypertrophic scar and its management are presented in this special issue. The article by van Zuijlen et al. [14] provided in-depth evaluation on not only skin substitutes, but also subcutaneous fat tissue and cartilage. While Chua and his colleagues [15] will guide us through the history of skin tissue engineering, from the initial development to up-to-dated advancement of dermal substitutes implementation methods available to clinicians. Through combining the knowledge together, we hope that we can offer a holistic picture of tissue engineering for practitioners working in the field of burn and scar management and promoting healing of wounds through skin tissue engineering.

In 2014, a national survey was conducted in China on burn rehabilitation services, in which several challenging issues facing China burn society were pointed out, highlighting the importance of professional development and specialist education [16]. With the aforementioned national guideline published, the Chinese Burn Society has no doubt striding out the first step. Hopefully, this special issue will further enhance the reader’s understanding on the most updated scar management practice, thus inspiring further research in management of hypertrophic scar. Our vision is first to create a “scar-less world”, then to the “scarless world”.
